# Hepatolithiasis After Living Donor Liver Transplantation in Pediatric Patients: Mechanism, Diagnosis, Treatment, and Prognosis

**DOI:** 10.3389/ti.2022.10220

**Published:** 2022-02-14

**Authors:** Yukihiro Sanada, Yasunaru Sakuma, Yasuharu Onishi, Noriki Okada, Yuta Hirata, Toshio Horiuchi, Takahiko Omameuda, Alan Kawarai Lefor, Naohiro Sata

**Affiliations:** Division of Gastroenterological, General and Transplant Surgery, Department of Surgery, Jichi Medical University, Shimotsuke, Japan

**Keywords:** hepatolithiasis, pediatric living donor liver transplantation, percutaneous transhepatic biliary drainage, double-balloon enteroscopy, computed tomography scan

## Abstract

There is little information about the outcomes of pediatric patients with hepatolithiasis after living donor liver transplantation (LDLT). We retrospectively reviewed hepatolithiasis after pediatric LDLT. Between May 2001 and December 2020, 310 pediatric patients underwent LDLT with hepaticojejunostomy. Treatment for 57 patients (18%) with post-transplant biliary strictures included interventions through double-balloon enteroscopy (DBE) in 100 times, percutaneous transhepatic biliary drainage (PTBD) in 43, surgical re-anastomosis in 4, and repeat liver transplantation in 3. The median age and interval at treatment were 12.3 years old and 2.4 years after LDLT, respectively. At the time of treatments, 23 patients (7%) had developed hepatolithiasis of whom 12 (52%) were diagnosed by computed tomography before treatment. Treatment for hepatolithiasis included intervention through DBE performed 34 times and PTBD 6, including lithotripsy by catheter 23 times, removal of plastic stent in 8, natural exclusion after balloon dilatation in 7, and impossibility of removal in 2. The incidence of recurrent hepatolithiasis was 30%. The 15-years graft survival rates in patients with and without hepatolithiasis were 91% and 89%, respectively (*p* = 0.860). Although hepatolithiasis after pediatric LDLT can be treated using interventions through DBE or PTBD and its long-term prognosis is good, the recurrence rate is somewhat high.

## Graphical Abstract

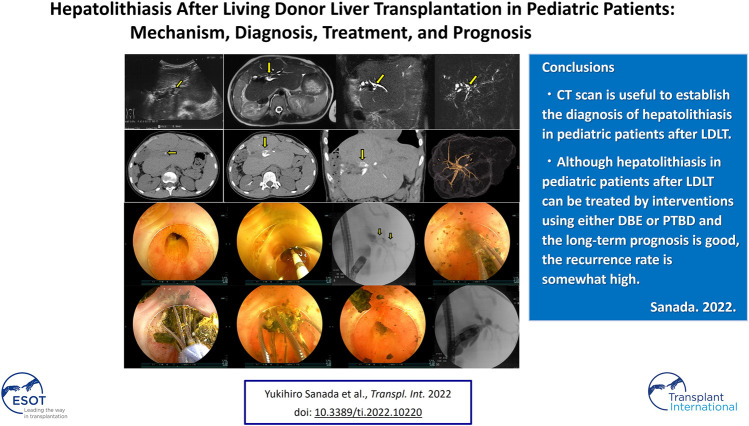

## Introduction

Liver transplantation (LT) is an established curative treatment for pediatric patients with end-stage liver disease or acute liver failure. However, post-transplant biliary complications are still frequent despite improvements and innovations in surgical techniques, and these complications occasionally lead to graft failure or even death. The reported incidence of biliary complications after living donor liver transplantation (LDLT) is 10–35% in pediatric recipients (1–6). However, hepatolithiasis after LT has been rarely reported. The reported incidence of hepatolithiasis or biliary cast syndrome after LT is 2.1–9.1% in adult recipients (7–10). The suggested risk factors for hepatolithiasis or biliary cast syndrome after LT include acute cellular rejection, prolonged warm ischemic time, and others (7–10). Few studies have analyzed the risk factors for hepatolithiasis after LT in pediatric recipients.

There are currently two major therapeutic options for biliary complications: surgical and non-surgical interventions. Non-surgical interventions, including percutaneous transhepatic biliary drainage (PTBD) and endoscopic interventions, have emerged as an attractive and less invasive alternatives to surgical intervention in recent years (2, 3). Endoscopic interventions remain controversial in pediatric recipients with a Roux-en-Y hepaticojejunostomy due to the presence of abdominal adhesions, the pediatric physique, and uncertain long-term patency. We reported that endoscopic interventions through double-balloon enteroscopy (DBE) for biliary strictures in pediatric recipients with Roux-en-Y hepaticojejunostomy after LDLT is safer and less invasive than surgical interventions (6). Few studies have analyzed the treatment options for hepatolithiasis after LT in pediatric recipients, and no consensus regarding the optimal approach has yet been reached.

We retrospectively reviewed the mechanism, diagnosis, treatment options and prognosis for pediatric patients with hepatolithiasis after LDLT.

## Materials and Methods

### Patients

Between May 2001 and December 2020, 314 LDLTs were performed for pediatric patients with end-stage liver disease or acute liver failure at the Department of Surgery, Division of Gastroenterological, General and Transplant Surgery, Jichi Medical University, Japan. Of these, four patients underwent LDLT with a choledochocholedochostomy; these patients were excluded from this study. Therefore, a total of 310 LDLTs with a hepaticojejunostomy were reviewed in the present study. Demographic data for recipients and graft information are shown in Table 1. Approval to conduct this study was obtained from the Ethics Committees of Jichi Medical University (Ethics Committee Approval Case Number 20-001).

**TABLE 1:** Demographic data for recipients and graft information.

Patient	Recipients with hepatolithiasis	Recipients without hepatolithiasis	*p*-value
Period	May 2001–December 2020		
Number	23	287	
Gender	Male: 11, Female: 12	Male: 107, Female: 180	0.374
Age (years old)	1.8 (0.6–16.0) years old	1.4 (0.0–16.5) years old	0.191
Weight	11.1 (5.8–64.9) kg	9.7 (2.6–62.9) kg	0.108
Original disease	Biliary atresia: 19,	Biliary atresia: 202,	
	OTCD: 2,	OTCD: 17,	
	Wilson’s disease: 1,	Graft failure: 12,	
	Primary sclerosing cholangitis: 1	Alagille syndrome: 11,	
		Acute liver failure: 7	
		Hepatoblastoma: 5,	
		Neonatal hemochromatosis: 5,	
		Others: 28	
ABO-compatibility	Identical/Compatible: 20,	Identical/Compatible: 235,	0.777
	Incompatible: 3	Incompatible: 52	
PELD/MELD score	12 (0–26)	9 (0–37)	0.304
Type of graft	Left lateral segment: 13,	Left lateral segment: 193,	
	Left lobe: 6,	Left lobe: 57,	
	Left lobe + caudate lobe: 3,	Reduced left lateral segment: 14,	
	Reduced left lateral segment: 1	Segment 2 monosegment: 13,	
		Left lobe + caudate lobe: 8,	
		Segment 3 monosegment: 1,	
		Posterior segment: 1	
GV/SLV	67.1 ± 25.4%	72.1 ± 20.0%	0.239
Operation time	15 hr38 min ± 5 hr03 min	14 hr30 min ± 4 hr34 min	0.244
Cold ischemic time	2 hr27 min ± 1 hr 29min	2 hr12 min ± 1 hr44 min	0.193
Warm ischemic time	54 min ± 24 min	52 min ± 19 min	0.959
Bleeding volume	78.7 ± 56.1 ml/kg	106.8 ± 124.3 ml/kg	0.837
Transfusion volume	102.7 ± 90.9 ml/kg	135.0 ± 143.3 ml/kg	0.297
Observation period	10.3 ± 5.6 years		

OTCD; ornithine transcarbamylase deficiency, PELD; pediatric end-stage liver disease, MELD; model for end-stage liver disease, GV/SLV; graft volume/standard liver volume ratio.

### Surgical Procedure of LDLT

The type of donor hepatectomy was selected based on the recipient’s standard liver volume, weight and graft volume determined by preoperative computed tomographic volumetry. The donor’s biliary anatomy was evaluated using intraoperative real-time cholangiography performed three times to determine the biliary anatomy, decide on the biliary transection line, and confirm absence of biliary leakage. A routine donor hepatectomy was performed using intraoperative ultrasonic guidance. The donor’s left hilar plate was transected using a scalpel.

For the recipient’s operation, inverted T-shaped or transverse incisions was made, and total hepatectomy was performed. In many infants, after total hepatectomy, the recipient’s right, middle and left hepatic veins were formed into a single orifice, and the recipient’s hepatic vein was anastomosed to the graft’s hepatic vein. The recipient’s portal vein was anastomosed to the graft’s left portal vein. Hepatic artery reconstruction was performed using microsurgical techniques. Biliary reconstruction was performed using a Roux-en-Y hepaticojejunostomy which was performed using intraluminal continuous 6-0 absorbable monofilament sutures on the posterior wall and extraluminal interrupted 6-0 absorbable monofilament sutures under surgical loupe vision. Recipients who underwent LDLT between May 2001 and March 2004, and between July 2008 and May 2011 underwent hepaticojejunostomy using an internal stent (4 or 5 Fr pancreatic duct tube, Sumitomo Bakelite co., Ltd., Tokyo, JAPAN, or 10 Fr Blake silicone drains, Johnson & Johnson, Tokyo, JAPAN), while recipients who underwent LDLT between April 2004 and July 2008 underwent biliary reconstruction without a stent. Recipients who underwent LDLT from June 2011 onwards underwent hepaticojejunostomy using an external stent (4 Fr pancreatic duct tube, Sumitomo Bakelite co., Ltd., Tokyo, JAPAN). If drained bile volume decreased to less than 50 ml/day without liver dysfunction, clamping of the external stent without cholangiography was considered. The external stent was removed 3 months after LDLT without cholangiography.

### Diagnosis of Post-Transplant Biliary Complications

We diagnosed post-transplant biliary complications including hepatolithiasis when radiologic, endoscopic, or surgical interventions were performed for patients with liver dysfunction or cholangitis due to biliary complications detected by ultrasonography or computed tomography (CT) scan. Obstruction at the biliary stricture site was diagnosed when contrast medium delivered via PTBD did not flow into the Roux-en-Y limb, when no real-time moving images were obtained under fluoroscopy, or when the hepaticojejunal anastomotic site could not be confirmed with DBE.

### Therapeutic Strategy for Biliary Complications

We present a summary of the therapeutic strategy for biliary complications including hepatolithiasis based on a previous report (6). When patients with suspected biliary complications experience persistent liver dysfunction or recurrent cholangitis, we assessed the hepaticojejunal anastomotic site using PTBD or DBE (EN-450P5/20 or EC-450BI5; Fujifilm Corp., Tokyo, Japan). The indication for DBE is weight greater than 15 kg because of instrument and technical limitations. When patients are diagnosed with biliary strictures by PTBD or DBE, balloon dilatation is performed. When obstruction of the hepaticojejunal anastomotic site or intrahepatic bile duct is diagnosed, balloon dilatation using the Rendezvous penetration method with DBE and PTBD is performed. If non-surgical interventions by balloon dilatation or the Rendezvous technique are unsuccessful, surgical re-anastomosis or repeat LT is performed.

### Statistical Analysis

Graft survival rate was calculated by the Kaplan-Meier product-limited method, and differences in survival between two groups then compared using the log-rank test. Statistical analysis was performed using the EZR (Saitama Medical Center, Jichi Medical University, Saitama, Japan), which is a graphical user interface for R (The R Foundation for Statistical Computing, Vienna, Austria), and differences were considered to be significant with values of *p* < 0.05.

## Results

The overall incidence of post-transplant biliary strictures was 18% (57/310). Treatment for patients with post-transplant biliary strictures included interventions through DBE in 100 patients, interventions after PTBD in 43, surgical re-anastomosis in 4, and repeat LT in 3. The median age and post-transplant interval at treatment were 12.3 years old (range 0.7–25.8) and 2.3 years after LDLT (range 0.1–19.3), respectively.

**FIGURE 1:**
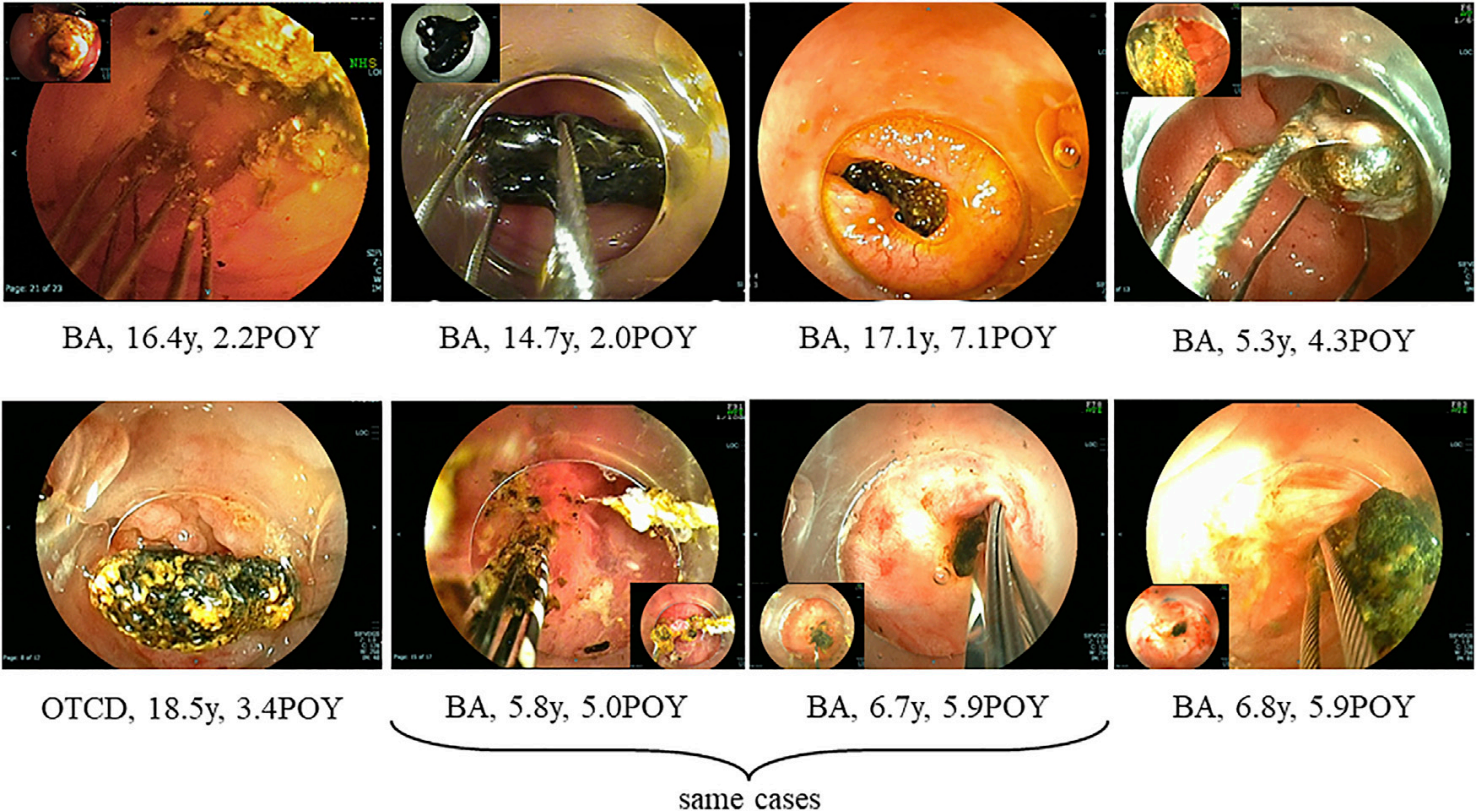
Types of hepatolithiasis diagnosed by computed tomography scan before treatment for post-transplant anastomotic biliary strictures. BA; biliary atresia, POY; post-operative year, OTCD; ornithine transcarbamylase deficiency.

**FIGURE 2:**
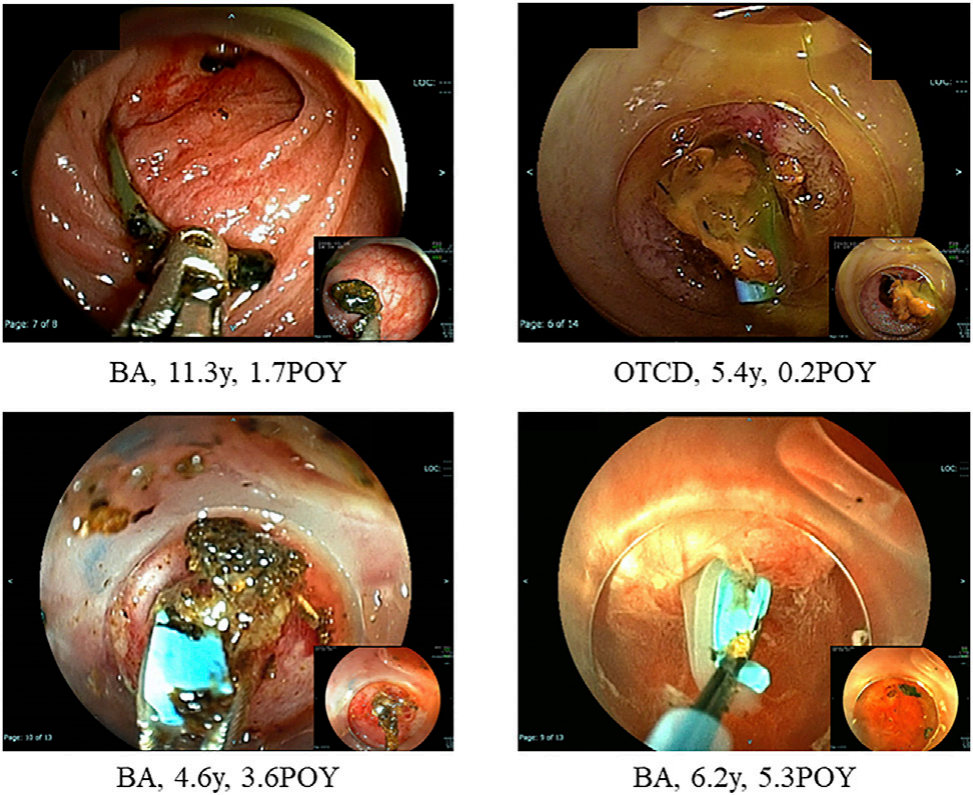
Hepatolith adhering to an internal stent at the time of living donor liver transplantation. BA; biliary atresia, POY; post-operative year, OTCD; ornithine transcarbamylase deficiency.

**FIGURE 3:**
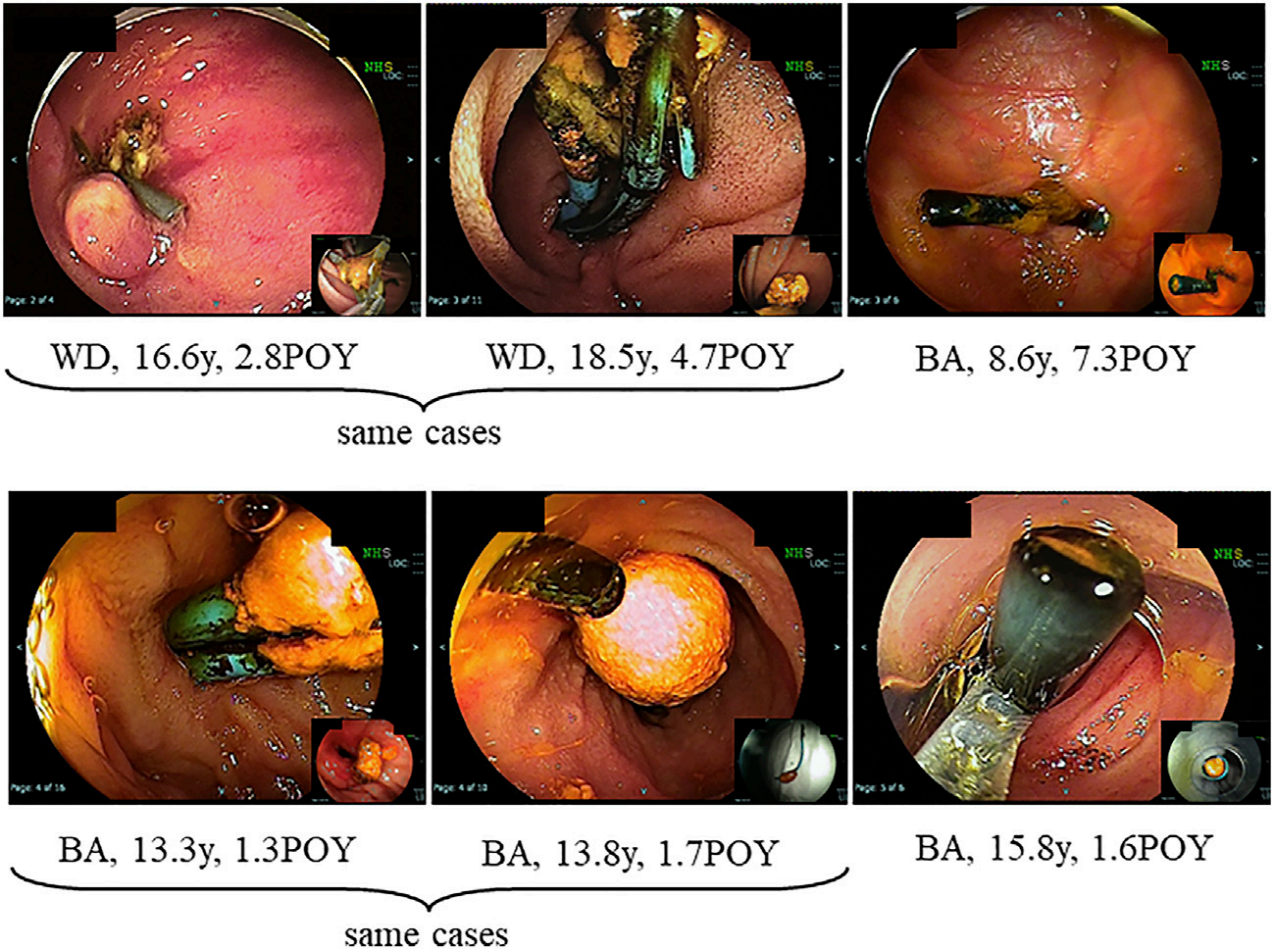
Hepatolith adhering to a plastic stent after treatment for post-transplant anastomotic biliary stricture. WD; Wilson’s disease, POY; post-operative year, BA; biliary atresia.

**FIGURE 4:**
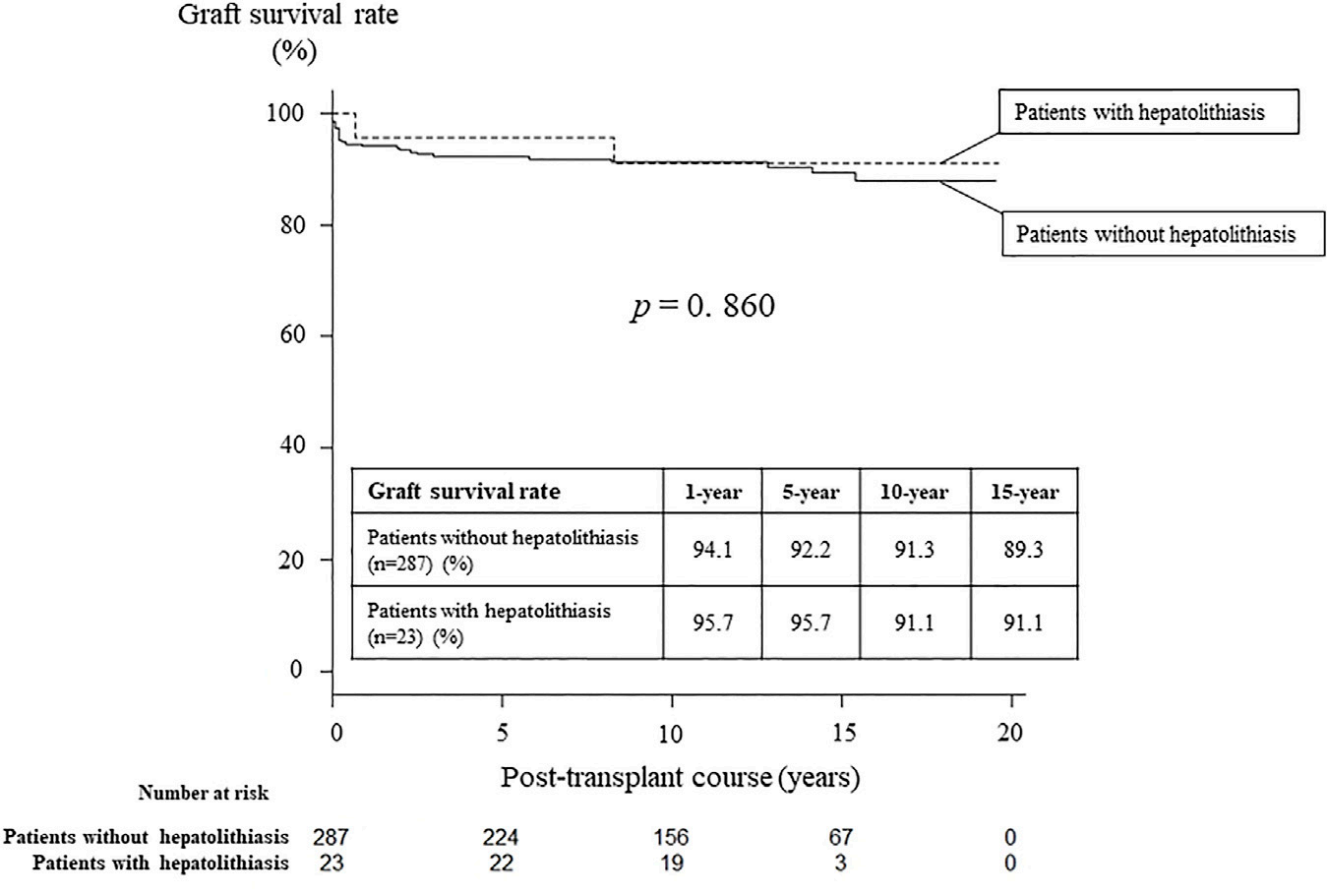
Graft survival rates in patients with and without hepatolithiasis after living donor liver transplantation.

At treatments, 23 patients (7%) had hepatolithiasis of whom 12 patients (52%) were diagnosed by CT scan before treatment (Figure 1). The hepatoliths were all calcium bilirubinate calculi. Treatment for hepatolithiasis included interventions through DBE in 34 times and interventions through PTBD in 6 times, including lithotripsy by catheter 23 times, removal of the plastic stent in 8 (Figures 2, 3), natural exclusion after balloon dilatation in 8, and impossibility of removal in 2. The incidence of recurrent hepatolithiasis was 30%, and repeat treatment was performed multiple times (range 2–6). The 15-years graft survival rates in patients with and without hepatolithiasis after LDLT were 91% and 89%, respectively (*p* = 0.860) (Figure 4), and the causes of graft failure included antibody-mediated rejection due to ABO-incompatible LDLT and chronic rejection. There was no significant difference in the rate of post-transplant complications between patients with and without hepatolithiasis (Table 2).

**TABLE 2:** Univariate analysis of risk factors for post-transplant complications in recipients with and without hepatolithiasis.

Variable	Recipients with hepatolithiasis N = 23	Recipients without hepatolithiasis N = 287	*p*-value
Hepatic vein complications	0 (0%)	24 (8.4%)	0.236
Portal vein complications	5 (21.7%)	41 (14.3%)	0.358
Hepatic artery complications	2 (8.7%)	15 (5.2%)	0.365
Re-laparotomy after LDLT	4 (17.4%)	33 (11.5%)	0.498
Acute cellular rejection	11 (47.8%)	115 (40.1%)	0.512
Steroid-resistant acute rejection	2 (8.7%)	32 (11.1%)	0.999
Cytomegalovirus viremia	9 (39.1%)	106 (36.9%)	0.826
Post-transplant lymphoproliferative disorder	1 (4.3%)	5 (1.7%)	0.373
Hospital length of stay	43 ± 28 days	47 ± 46 days	0.501

LDLT; living donor liver transplantation.

### Presentation of a Patient With Hepatolithiasis

A female with biliary atresia underwent LDLT using a left lateral segment graft from her mother at age 0.8 years. Hepatic arterial thrombosis developed on post-operative days 4, 7, and 17. She underwent percutaneous transfemoral artery balloon dilatation on each occasion. Non-anastomotic biliary strictures developed on post-operative days 36 and 50. She underwent PTBD on each occasion. Thereafter, she has suffered from repeat episodes of cholangitis and mild liver dysfunction.

**FIGURE 5:**
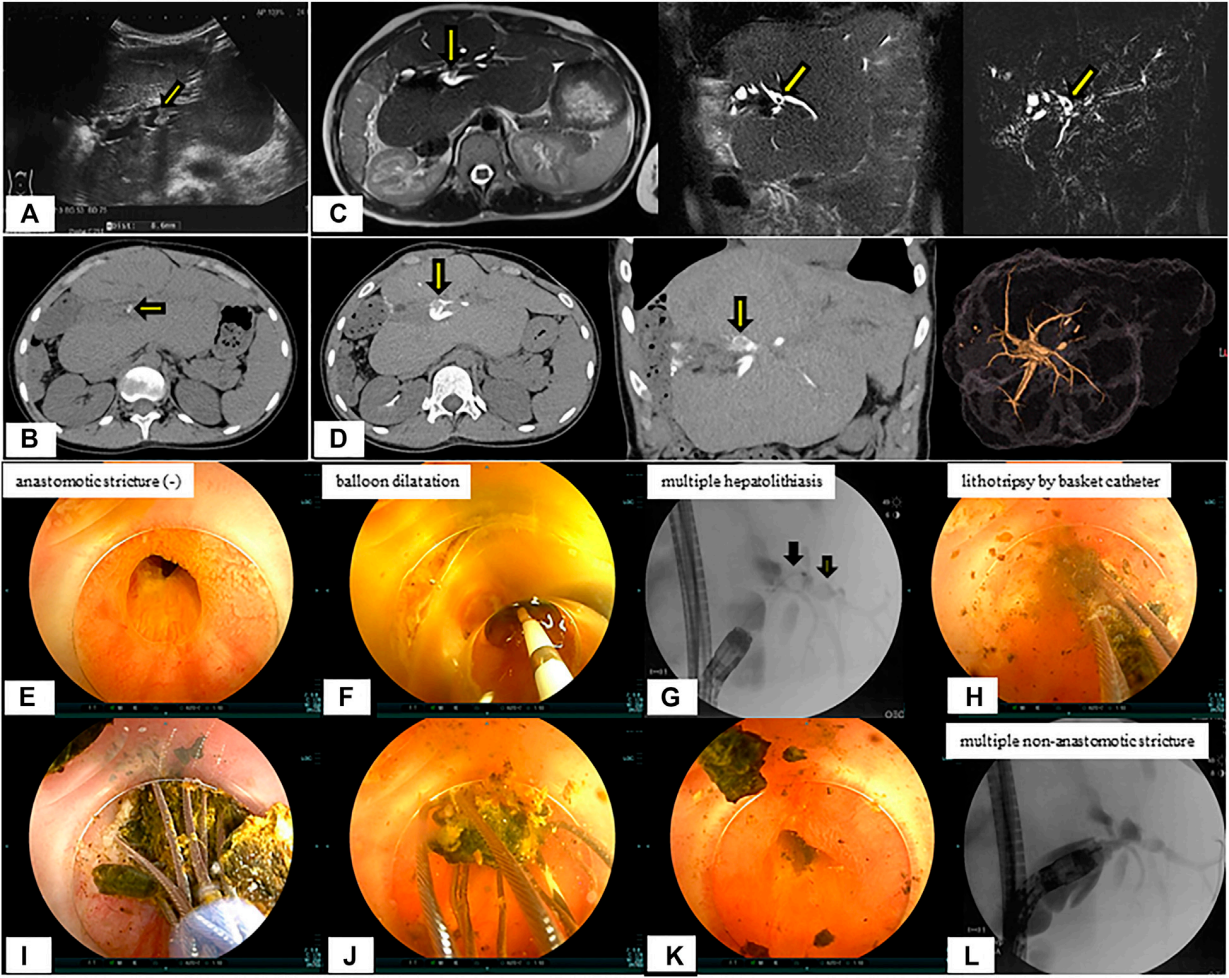
Radiological findings and endoscopic lithotripsy for hepatolithiasis in a patient with multiple non-anastomotic biliary strictures. **(A)** Hepatolith (diameter 8.6 mm) with acoustic shadowing on ultrasonography was observed at the confluence of the biliary tracts of Segments 2 and 3. **(B)** Hepatolithiasis with a high density lesion on computed tomography scan was observed at the confluence of the biliary tracts of Segments 2 and 3. **(C)** Magnetic resonance imaging. **(D)** Drip infusion cholangiographic-computed tomography. **(E)** Anastomotic biliary stricture was not observed by double-balloon enteroscopy. **(F)** Balloon dilatation for intrahepatic bile duct stricture was performed. **(G)** Multiple hepatoliths were demonstrared on cholangiography at the confluence of the biliary tracts of Segments 2 and 3. **(H–K)** Lithotripsy by basket catheter was performed. **(L)** Multiple intrahepatic bile duct strictures on cholangiography were observed and hepatoliths disappeared.

After developing acute cholangitis at age 15.3 years, she was diagnosed with hepatolithiasis by imaging studies (Figures 5A–D). She was diagnosed with hepatolithiasis with a non-anastomotic biliary stricture by direct vision and cholangiography using DBE. Lithotripsy by basket catheter was performed (Figures 5E–L). Although she had a small hepatolith a year after treatment, she is doing well without further episodes of cholangitis.

## Discussion

Hepatolithiasis after LT has been rarely reported. The reported incidence of hepatolithiasis or biliary cast syndrome after LT is 2.1–9.1% in adult recipients (7–10). However, few studies have analyzed the incidence of hepatolithiasis after LT in pediatric recipients. Therefore, no consensus on the optimal diagnostic or treatment strategies has been reached, and the prognosis of hepatolithiasis after LT in pediatric recipients is not defined. The suggested risk factors for hepatolithiasis or biliary cast syndrome after LT include acute cellular rejection, prolonged warm ischemic time, and others in adult recipients (7–10). In this study, the suggested risk factors for hepatolithiasis after LDLT in pediatric recipients made clear hepaticojejunostomy, internal stent placed during LDLT, plastic stent placed after treatment for post-transplant anastomotic biliary stricture, and non-anastomotic biliary stricture. The hepatoliths were all calcium bilirubinate calculi. Therefore, reflux of intestinal juice via hepaticojejunostomy, adhesion to an internal stent placed during LDLT, a plastic stent placed after treatment for post-transplant anastomotic biliary stricture (Figures 2, 3), and biliary stasis due to non-anastomotic biliary strictures were associated with the development of hepatolithiasis. In our institution, recipients who underwent LDLT after June 2011 underwent hepaticojejunostomy using an external stent. Thereafter, the incidence of post-transplant anastomotic biliary strictures was significantly decreased. After this, patients with adhesion to an internal stent placed at LDLT or a plastic stent placed after treatment for post-transplant anastomotic biliary strictures should be decreasing, and therefore, the incidence of hepatolithiasis is also expected to decrease. On the other hands, the causes and prognosis of post-transplant non-anastomotic biliary strictures are unclear, but in adult recipients, it has been reported that the incidence of repeat LT and mortality was high because it is difficult to treat non-anastomotic biliary strictures and to resolve the cause of non-anastomotic biliary strictures (11, 12). In this study, one patient after lithotripsy for hepatolithiasis developed intractable cholangitis with repeat hepatolithiasis. The long-term prognosis of patients with post-transplant non-anastomotic biliary strictures is not defined, and patients with post-transplant non-anastomotic biliary strictures may eventually need repeat LT.

We diagnosed post-transplant biliary complications including hepatolithiasis when radiologic, endoscopic, or surgical interventions were performed for the patients with liver dysfunction or cholangitis due to biliary complications detected by ultrasonography or CT scan (Figure 5B). Hepatolithiasis was diagnosed in 12 patients (52%) by CT scan before treatment was performed (Figure 1). Indwelling internal stents and long-term indwelling plastic stents should be noted, and CT scan is useful to establish the diagnosis of hepatolithiasis in these patients after LDLT in pediatric patients.

There are currently two major therapeutic options for patients with biliary complications that can be classified as surgical and non-surgical interventions. Non-surgical interventions, including PTBD or endoscopic interventions, have emerged as an attractive and less invasive alternative to surgical interventions in recent years (2, 3). We have reported that endoscopic interventions through DBE to evaluate and treat biliary strictures in pediatric patients with Roux-en-Y hepaticojejunostomies after LDLT is safer and less invasive than surgical interventions (6). Although few studies have analyzed treatment options for pediatric patients with hepatolithiasis after LT, with advances in endoscopic instrumentation and techniques in recent years, endoscopic treatment of hepatolithiasis using DBE has become possible. Therefore, in our institution, the first-line treatment for post-transplant biliary complications including hepatolithiasis in pediatric patients with a Roux-en-Y hepaticojejunostomy is endoscopic intervention using DBE. However, in this study, the incidence of recurrent hepatolithiasis was 30%, and treatment was repeated multiple times (range 2–6). This recurrence rate of hepatolithiasis is thought to be associated with the use of endoscopic interventions using DBE.

In conclusion, mechanisms causing hepatholitiasis following pediatric LDLT and also preventive measures were made clear in this study. In addition, diagnostic methods and treatment options for hepatolithiasis following pediatric LDLT were showed for the first time. CT scan is useful to establish the diagnosis of hepatolithiasis in pediatric patients after LDLT. Although hepatolithiasis in pediatric patients after LDLT can be treated by interventions using either DBE or PTBD and the long-term prognosis is good, the recurrence rate is somewhat high. Further studies of our policy for the diagnosis and treatment of hepatolithiasis after LDLT and the accumulation more experience are necessary.

## Capsule Sentence Summary

The overall incidence of hepatolithiasis after pediatric living donor liver transplnatation was 7% (23/310). Although hepatolithiasis in pediatric patients can be treated by interventions using either double-balloon enteroscopy or percutaneous transhepatic biliary drainage and the long-term prognosis is good, the recurrence rate is somewhat high.

## Data Availability

The datasets presented in this study can be found in online repositories. The names of the repository/repositories and accession number(s) can be found in the article/supplementary material.

## Abbreviations

LT, liver transplantation; LDLT, living donor liver transplantation; PTBD, percutaneous transhepatic biliary drainage; DBE, double-balloon enteroscopy; CT, computed tomography.

## References

[1] Darius T Rivera J Fusaro F Lai Q de Magnée C Bourdeaux C Risk Factors and Surgical Management of Anastomotic Biliary Complications after Pediatric Liver Transplantation. Liver Transpl (2014) 20(8):893–903. 10.1002/lt.23910 24809592

[2] Feier FH Chapchap P Pugliese R da Fonseca EA Carnevale FC Moreira AM Diagnosis and Management of Biliary Complications in Pediatric Living Donor Liver Transplant Recipients. Liver Transpl (2014) 20(8):882–92. 10.1002/lt.23896 24760734

[3] Chok KSH Chan SC Chan KL Sharr WW Tam PKH Fan ST Bile Duct Anastomotic Stricture after Pediatric Living Donor Liver Transplantation. J Pediatr Surg (2012) 47(7):1399–403. 10.1016/j.jpedsurg.2011.12.014 22813803

[4] Lüthold SC Kaseje N Jannot AS Mentha G Majno P Toso C Risk Factors for Early and Late Biliary Complications in Pediatric Liver Transplantation. Pediatr Transpl (2014) 18(8):822–30. 10.1111/petr.12363 25263826

[5] Feier FH Seda-Neto J da Fonseca EA Candido HLL Pugliese RS Neiva R Analysis of Factors Associated with Biliary Complications in Children after Liver Transplantation. Transplantation (2016) 100(9):1944–54. 10.1097/tp.0000000000001298 27362317

[6] Sanada Y Katano T Hirata Y Yamada N Okada N Ihara Y Biliary Complications Following Pediatric Living Donor Liver Transplantation: Risk Factors, Treatments, and Prognosis. Transplantation (2019) 103(9):1863–70. 10.1097/tp.0000000000002572 30720679

[7] Gor NV Levy RM Ahn J Kogan D Dodson SF Cohen SM Biliary Cast Syndrome Following Liver Transplantation: Predictive Factors and Clinical Outcomes. Liver Transpl (2008) 14(10):1466–72. 10.1002/lt.21492 18825683

[8] Zhu X-d. Shen Z Shen Z-y. Chen X-g. Zang Y-j. Pathotyping and Clinical Manifestations of Biliary Cast Syndrome in Patients after an Orthotopic Liver Transplant. Exp Clin Transpl (2013) 11(2):142–9. 10.6002/ect.2012.0035 23190361

[9] Paik WH Lee SH Ryu JK Song BJ Kim J Kim Y-T Long-term Clinical Outcomes of Biliary Cast Syndrome in Liver Transplant Recipients. Liver Transpl (2013) 19(3):275–82. 10.1002/lt.23589 23213039

[10] Voigtländer T Negm AA Strassburg CP Lehner F Manns MP Lankisch TO. Biliary Cast Syndrome Post-Liver Transplantation: Risk Factors and Outcome. Liver Int (2013) 33(8):1287–92. 10.1111/liv.12181 23601581

[11] Nakamura N Nishida S Neff GR Vaidya A Levi DM Kato T Intrahepatic Biliary Strictures without Hepatic Artery Thrombosis after Liver Transplantation: an Analysis of 1,113 Liver Transplantations at a Single center. Transplantation (2005) 79(4):427–32. 10.1097/01.tp.0000152800.19986.9e 15729168

[12] Lee HW Suh K-S Shin WY Cho E-H Yi N-J Lee JM Classification and Prognosis of Intrahepatic Biliary Stricture after Liver Transplantation. Liver Transpl (2007) 13(12):1736–42. 10.1002/lt.21201 18044761

